# Interaction of *Veratrum nigrum* with *Panax ginseng* against Obesity: A Sang-ban Relationship

**DOI:** 10.1155/2013/732126

**Published:** 2013-09-02

**Authors:** Jinbong Park, Yong-Deok Jeon, Hye-Lin Kim, Hara Lim, Yunu Jung, Dong-Hyun Youn, Mi-Young Jeong, Hyun-Ju Kim, Sung-Hoon Kim, Su-Jin Kim, Seung-Heon Hong, Jae-Young Um

**Affiliations:** ^1^College of Korean Medicine, Institute of Korean Medicine, Kyung Hee University, 26 Kyungheedae-ro, Dongdaemun-Gu, Seoul 130701, Republic of Korea; ^2^Department of Oriental Pharmacy, College of Pharmacy, Wonkwang University, 460 Iksandae-ro, Iksan, Jeonbuk 570749, Republic of Korea; ^3^Department of Cosmeceutical Science, Daegu Haany University, 1 Haanydaero, Gyeongsan-si, Gyeongsangbuk-Do 712715, Republic of Korea

## Abstract

Obesity has become a major health threat in developed countries. However, current medications for obesity are limited because of their adverse effects. Interest in natural products for the treatment of obesity is thus rapidly growing. Korean Medicine (KM) is characterized by the wide use of herbal formulas. However, the combination rule of herbal formulas in KM lacks experimental evidence. According to *Shennong's Classic of Materia Medica*, the earliest book of herbal medicine, *Veratrum nigrum* (VN) has antagonistic features against *Panax ginseng* (PG), and the PG-VN pair is strictly forbidden. In this study, we have shown the effects of PG, VN, and their combination on obesity in high-fat (HF) diet-induced obese mice and in 3T3-L1 cells. PG, VN, and PG-VN combination significantly reduced weight gain and the fat pad weight in HF diet-induced obese mice. They also significantly decreased lipid accumulation and the expressions of two major adipogenesis factors, PPAR**γ** and C/EBP**α**, in 3T3-L1 cells. In addition, the PG-VN combination had synergistic effects compared with the mixture of extracts of PG and VN on inhibition of PPAR**γ** and C/EBP**α** expressions at lower doses. These results indicate a new potential anti-obese pharmacotherapy and also provide scientific evidence supporting the usage of herbal combinations instead of mixtures in KM.

## 1. Introduction

Obesity is a public health dilemma, especially in developed countries, and has steadily increased in recent years. The World Health Organization currently estimates that over 1 billion individuals worldwide are overweight. Almost one-third of these individuals are clinically obese, markedly raising their chances of suffering cardiovascular disease, type 2 diabetes, cancer, and stroke [1]. Over 1 billion adults are either overweight BMI > 25 or obese BMI > 30, and even more problematic is that about 25% of children in the USA are also now overweight or obese. These numbers are expected to increase by more than half again by the year 2025 worldwide, with especially severe impact in less developed countries [2]. The regulation of body fat content in animals results from the integration of multiple nutrient, sensory, and hormonal inputs, primarily at the level of the brain and adipose tissues [3]. The mechanisms underlying the development of obesity may include changes in skeletal muscle and adipose tissue enzymatic and/or receptor regulation (lipoprotein lipase, hormone sensitive lipase, and very low-density lipoprotein receptor) and/or hormonal regulation (i.e., insulin, growth hormone, catecholamine), resulting from physical inactivity and/or inappropriate macronutrient intake (i.e., high levels of saturated fat and/or refined carbohydrates) [4]. Thus, the integrative network of obesity, a complex, systemslevel disease, is influenced not only by genetics but also by circadian rhythm and physical and social environments [5].

The transcriptional regulation of adipocyte differentiation is known to rely on interplays between several adipogenic transcription factors. Among these, the peroxisome proliferator-activated receptor gamma (PPAR*γ*) acts as a key regulator, both *in vitro* and *in vivo*, of the development of adipocytes and the only factor that can induce the adipocyte-like phenotype in nonadipogenic cell types [6]. The function of PPAR*γ* is relatively close to members of the CCAAT-enhancer-binding protein (C/EBP) family, which have important functions at different time points during adipogenesis. C/EBP*β* and C/EBP*δ* are expressed already in preadipocytes and are rapidly induced further [7], posttranslationally activated [8] by the adipogenic inducers cocktail. The expression of C/EBP*β* is induced by the cAMP response element-binding protein and the glucocorticoid receptor (GR), and the phosphorylation and activation of C/EBP*β* are induced by the extracellular-signal-regulated kinase 1/2 pathway [8]. On the other hand, C/EBP*δ* is induced by GR [9]. Both C/EBP*β* and C/EBP*δ* are direct activators of the expression of PPAR*γ* and C/EBP*α* [7, 10], the two major late-acting adipogenic transcription factors. These two factors are known to mutually induce the expression of each other and also cooperate to activate the adipogenesis gene program [11].

In contrast, according to “Huangdi's Internal Classic,” one of the oldest Oriental Medicine's classics that describes the most basic theories of Oriental Medicine, obesity is a result of overeating [12]. In Korean Medicine (KM)'s most well-known classic *Treasured Mirror of Eastern Medicine*, obesity is explained in a similar manner to the description in *Huangdi's Internal Classic*, as it refers to obesity as a result of food overwhelming one's original qi and it notes that it can shorten one's life [13].

The root of *Panax ginseng *C. A. Meyer (Radix Ginseng) is one of the most popular Korean herbal medicines and has also gained popularity in Western countries recently [14]. In KM, *Panax ginseng* (PG) is known to improve “well-being” by highly restoring and enhancing qi and fluid at the same time, acting like a tonic, rather than a curative medication for specific types of patients. Modern therapeutic claims refer to vitality, immune function, cancer, and improvement of cognitive and physical performance and sexual function [15]. In dietary obese mice, ethanol extract of wild PG significantly inhibited body weight gain, decreased blood glucose, triglycerides, and free fatty acids levels, and improved insulin sensitivity [16]. Efficacy of PG on glucose metabolism has been confirmed in patients with type 2 diabetes also, showing reduced fasting blood glucose and body weight [17]. PG was also shown to regulate lipid metabolism. The lipid metabolism by PG was reported 30 years ago, in a chicken *in vivo* model [18]. In a clinical trial, PG extract administration reduced total cholesterol, triglycerides, and LDL and induced HDL [19]. In addition to these features, ginseng has been widely studied for treatment of diabetes, dyslipidemia, and obesity. Its berries and leaves were also demonstrated to reduce blood glucose in diabetic models [20].

The root of *Veratrum nigrum* Linné var.* ussuriense *Loes. fil. (Radix Veratri nigri), commonly known as Black False Hellebore, is a coarse, highly poisonous perennial herb native to Asia and Europe [21]. *Veratrum nigrum* (VN) is known to be highly toxic [22], and due to its ability to cause nausea and vomiting, it is applied to dyspnea in epilepsy or stroke patients. Studies have revealed that VN is a potential agonist of *β*2-adrenoceptor [23] and can decrease blood pressure and heart rate in a dose-dependent manner in hypertensive rats [24], and it affords significant protection against hepatic ischemia/reperfusion injury in rats [25]. However, to date, no study of VN on obesity has been reported.

According to *Shennong's Classic of Materia Medica*,   VN has antagonistic features against PG [26]. This antagonism is one of the six types of “Chil-jeong.” The “Chil-jeong” theory first appeared in *Shennong's Classic of Materia Medica*, a classic of oriental medicine that introduced 365 kinds of herbs and described their qi and flavors, meridian entries, and special features [26]. The six types of “Chil-jeong” can be sorted into three kinds considering their major features. “Sang-soo” and “Sang-sa” are combinations where two herbs reinforce and have synergistic effects. On the other hand, “Sang-oe” and “Sang-swae” are types that can neutralize the toxicity of combinations. And finally, “Sang-o” and “Sang-ban” are types that are not recommended because of weakened effects or occurrences of adverse effects. A representative example of “Sang-ban” is a combination of VN and PG. This combination was introduced in *Shennong's Classic of Materia Medica* as one of the herbal pairs that should not be used together.

In this study, because the effects of PG and VN co-treatment on obesity have not been proved scientifically, the effects of PG and VN extracts on obesity are investigated both *in vivo* and *in vitro*, and an investigation of the effects of their combination is followed to search the antagonistic effects between the two and compare them with those of the separate extracts.

## 2. Materials and Methods

### 2.1. Preparation of PG, VN, and Their Combination

100 g of PG was chopped and soaked in 1000 mL of distilled water and extracted at 100°C for 3 hours. 100 g of VN was extracted by the same method. In order to prepare the combination extractions, 50 g of PG and 50 g of VN (1 : 1) or 90 g of PG and 10 g of VN (9 : 1) were extracted together by the same method. After filtering, the solvent was removed by evaporation. It was then freeze-dried to obtain the extracts. The samples were stored at −20°C.

### 2.2. Animal Experiments

Male C57BL/6J mice, weighing 17-18 g at the age of 4 weeks, were purchased from the Dae-Han Experimental Animal Center (Dae-Han Biolink, Eumsung, Republic of Korea). The animals were all maintained in conditions in accordance with the regulations issued by the Institutional Review Board of Kyung Hee University (confirmation number: KHUASP (SE)-13-012). The animals were housed under a 12-hour light/dark cycle at a humidity of 70% and a constant temperature of 23 ± 2°C. The animals were divided into six groups of five mice each. The six groups of mice were fed for 16 weeks with the following (1) a standard laboratory diet (CJ Feed Co., Ltd., Seoul, Korea); (2) a high-fat (HF) diet (60% fat); (3) a HF plus PG; (4) a HF plus VN; (5) a HF plus PGand VN combination; (6) a HF plus Slinti, a green tea extract used as a positive control (Myoungmoon Pharm. Co., LTD., Seoul, Republic of Korea). The components of the diets are described in Table 1. The diets were prepared according to the AIN-93G modified formulation. The animals were given free access to food and tap water. The body weight and food intake amount were recorded every week. At the end of this period, the animals were fasted overnight. The next day, they were anesthetized with ketamine and Rompun (5 : 3), and blood samples were collected by cardiac puncture. The organs and fat pads were immediately weighed.

### 2.3. Plasma Parameter Analysis

Plasma was separated immediately after blood sampling by centrifugation at 10,000 ×g for 10 min. Plasma concentrations of total cholesterol and high-density lipoprotein (HDL) cholesterol were determined using automated enzymatic methods [27], and low-density lipoprotein (LDL) cholesterol was calculated using the Friedewald formula [28]. The plasma concentration of triglyceride was measured enzymatically using a triglyceride assay kit (Asan Co., Seoul, Republic of Korea). Alanine aminotransferase (ALT) activity was determined with an ALT/GPT kit (Sigma-Aldrich, St. Louis, MO, USA).

### 2.4. Reagents

Dulbecco's modified Eagle's medium (DMEM), penicillin-streptomycin, fetal calf serum (FCS), and fetal bovine serum (FBS) were from Gibco BRL (Grand Island, NY). Insulin, 3-isobutylmethylxanthine (IBMX), and dexamethasone (DEX) were purchased from Sigma Chemical Co. (St. Louis, MO). Anti-C/EBP*α* and anti-GAPDH antibodies were purchased from Santa Cruz Biotechnology (Santa Cruz, CA). Anti-PPAR*γ* antibody was purchased from Cell Signaling Technology (Beverly, MA).

### 2.5. Cell Culture and Adipocyte Differentiation

3T3-L1 mouse embryo fibroblasts were obtained from the American Type Culture Collection (ATCC, Rockville, MD, USA). Cells were maintained in DMEM containing 10% FBS with 100 units/mL of penicillin-streptomycin solution at 37°C in 5% CO_2_ at 95% humidity until confluence. Two days after confluence (day 0), the cells were stimulated to differentiate with differentiation inducers (1 *μ*M dexamethasone, 500 *μ*M 3-isobutyl-1-methylxanthine, and 1 *μ*g/mL insulin) that were added to DMEM containing 10% FBS for 48 hours. On day 2, 3T3-L1 cells were then cultured in DMEM, 10% FBS supplemented with 1 *μ*g/mL insulin, for another two days. At that time, water extract of PG, water extract of VN, and water extract of their combination were prepared in the differentiation medium at concentrations of 0.01 mg/mL, 0.1 mg/mL, and 1 mg/mL. And then, the cells were cultured with 10% FBS/DMEM medium for an additional two days, at which time more than 90% of cells were mature adipocytes with accumulated fat droplets. On day 6, the cells were harvested and prepared for further experiments.

### 2.6. MTS Cell Viability Assay

Tests were performed in 96-well plates. 3T3-L1 preadipocytes were seeded (2 × 10^4^ cells/well) and incubated in 10% FBS/DMEM medium for 24 hours. The cells were then incubated in 10% FBS/DMEM medium containing water extract of PG, water extract of VN, and water extract of their combination for an additional 48 hours. Cell viability was monitored by a Cell Proliferation MTS Kit as recommended by the manufacturer [29]. Prior to measuring viability, treatment media were removed and replaced with 200 *μ*L of fresh 10% FBS/DMEM medium and 20 *μ*L of 3-(4,5-dimethylthiazol-2-yl)-5-(3-carboxymethoxyphenyl)-2-(4-sulfophenyl)-2H-tetrazolium (MTS) solution. Cells were then returned to the incubator for 4 hours. The absorbance was measured at 490 nm in a VERSAmax microplate reader (Molecular Devices, Sunnyvale, CA, USA) to determine the formazan concentration, which is proportional to the number of live cells.

### 2.7. Oil Red O Staining

Intracellular lipid accumulation was measured using Oil Red O. The Oil Red O working solution was prepared as described by Ramirez-Zacarias et al. [30]. 3T3-L1 adipocytes were harvested 6 days after the initiation of differentiation. Cells were washed twice with phosphate buffered saline (PBS, pH 7.4) and then fixed with 10% neutral formalin for 2 hours at room temperature. After washing with 60% isopropanol, the cells were stained with Oil Red O working solution for 30 min and then washed 4 times with water to remove the unbound dye. The stained cells were observed by an Olympus IX71 Research Inverted Phase microscope (Olympus Co., Tokyo, Japan). Following the microscopic observation, 100% isopropanol was added as an extraction solution to extract the staining dye of cells. The absorbance of the extracted dye was measured spectrophotometrically at 500 nm in a VERSAmax microplate reader (Molecular Devices, Sunnyvale, CA, USA). 

### 2.8. RNA Isolation and Real-Time Reverse-Transcription Polymerase Chain Reaction (RT-PCR)

Total cellular RNA was isolated from 3T3-L1 adipocytes using a GeneAll RiboEx Total RNA extraction kit (GeneAll Biotechnology, Seoul, Republic of Korea) and QIAzol lysis reagent (QIAZEN sciences, Maryland, USA). Total RNA was used as a template for first-strand cDNA synthesis performed using a Power cDNA Synthesis Kit (iNtRON Biotechnology, Seoul, Republic of Korea) according to the manufacturer's instructions. PCR products were measured with a StepOnePlus Real-time RT-PCR System (Applied Biosystems, Foster City, CA, USA), and the relative gene expression was calculated based on the comparative CT method using StepOne Software v2.1 (Applied Biosystems, Foster City, CA, USA). The expression of glyceraldehyde-3-phosphate dehydrogenase (GAPDH) mRNA was used as an endogenous control. The target cDNA was amplified using the sense and antisense primers described in Table 2.

### 2.9. Western Blot Analysis

After experimental treatment, cells were washed twice with ice-cold PBS and lysed with RIPA lysis buffer, which consisted of 50 mM Tris-HCl (pH 7.5), 0.1% sodium dodecyl sulphate (SDS), 0.1% Triton X-100, 1% Nonidet P-40, 0.5% sodium deoxycholate, 150 mM NaCl, and 1 mM phenylmethylsulfonyl fluoride. Insoluble materials were removed by centrifugation at 13,000 rpm for 20 min at 4°C. The total concentration of extracted proteins was determined using the method of Bradford [31]. The proteins in the supernatants were separated by 8% sodium dodecyl sulfate-polyacrylamide gel electrophoresis (SDS-PAGE) and transferred onto polyvinylidene difluoride (PVDF) membranes. After blocking with 10 mM Tris, 150 mM NaCl, and 0.05% Tween-20 (TBST) (pH 7.6) containing 5% skim milk for 1 hour at room temperature, the membranes were washed with TBST and then incubated with the appropriate primary antibodies against PPAR*γ* (Cell Signaling Technology, Beverly, MA, USA), C/EBP*α*, or GAPDH (Santa Cruz Biotechnology, Santa Cruz, CA, USA) at 4°C overnight. After washing with TBST, the blots were subsequently incubated with horseradish peroxidase (HRP)-conjugated AffiniPure goat anti-rabbit IgG or Goat anti-mouse IgG (Jackson Immunoresearch Lab., USA) in 5% skim milk TBST at room temperature for 1 h. Protein signals were developed by using the ECL Western Blotting Detection Reagent (Amersham Bioscience, Piscataway, NJ, USA). All experiments were repeated at least three times. Representative Western blots are shown along with graphs of the quantitative data. The chemiluminescent intensities of protein signals were quantified using Quantity One software (Bio-Rad Laboratories, Hercules, CA, USA). 

### 2.10. Statistical Analysis

Results are expressed as the mean ± SEM of independent experiments, and statistical analyses were performed using a Student's *t*-test to determine differences between groups. All statistical analyses were performed using SPSS statistical analysis software version 11.5 (SPSS Inc., Chicago, IL, USA). Values with *P < 0.05 were considered to indicate statistical significance.

## 3. Results

### 3.1. Effects of PG, VN, and Their Combination on Body Weight in HF Diet-Induced Obese C57BL/6J Mice

**Figure 1:**
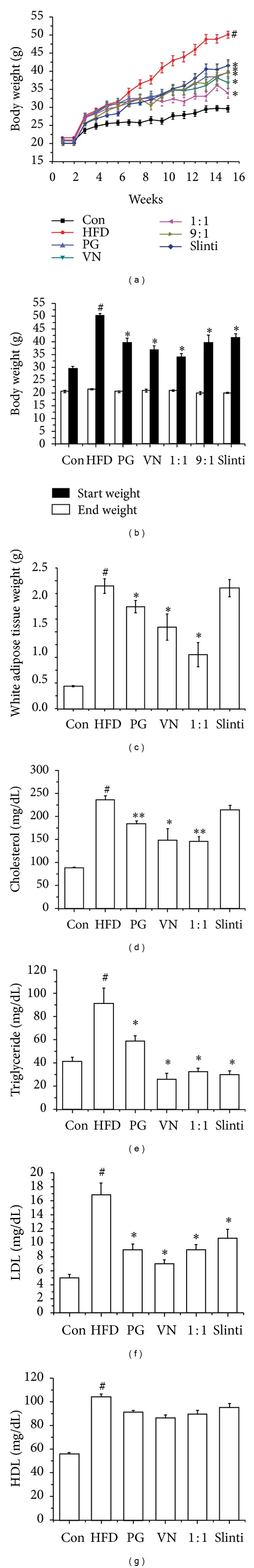
Effects of PG, VN, and their combinations in HF diet-induced obese mice. The weight changes of control, HF diet, PG, VN, PG-VN combination, and slinti groups were measured for 16 weeks (a). The weight difference between the start weight and end weight of each group was measured to evaluate the weight gain (b). The white adipose tissue weights (c), total cholesterol (d), triglyceride (e), LDL-cholesterol (f), and HDL-cholesterol (g) of the mice were also measured. All values are mean ± SD. ^#^ *P* < 0.05, significantly different from the Control; *P < 0.05, significantly different from the HF diet group. Con, standard laboratory diet group; HFD, high-fat diet group; PG, HF diet plus PG group; VN, HF diet plus VN group; 1 : 1, HF diet plus PG-VN 1 : 1 combination group; 9 : 1, HF diet plus PG-VN 9 : 1 combination group; Slinti, HF diet plus slinti group. PG, *Panax ginseng*; VN, *Veratrum nigrum. *

Experimental animals appeared healthy, showing no pathological signs or abnormalities during the feeding period. As shown in Figures 1(a) and 1(b), the seven groups had similar body weights at the beginning of the study. However, mice fed the HF diet gained significantly more weight than those fed the standard diet mice (*P* < 0.05). On the other hand, weight gain in the PG, VN, PG-VN 1 : 1 combination, and PG-VN 9 : 1 combination groups was significantly less than that in the HF diet group (*P* < 0.05). The weight gain in the PG-VN 1 : 1 combination group also significantly decreased compared with the PG group (*P* < 0.05), but not with the VN group. The HF diet group gained 27.49 ± 1.01 g of weight, while the PG group gained 18.07 ± 1.13 g, the VN group gained 15.06 ±1.07 g, the slinti (consisted of green tea powder 250 mg and orthosiphon powder 150 mg) group, which is the positive control group, gained 20.59 ± 1.25 g, and the PG-VN 1 : 1 combination group gained 12.17 ± 1.13 g of weight after 16 weeks. Antiobese effect of the PG-VN 1 : 1 combination group in weight gain was higher than that in the PG-VN 9 : 1 combination group. Therefore, further PG-VN combination studies were performed at the ratio of 1 : 1. Changes in the fat pads among the six groups are given in Figure 1(c). The results showed significant differences in the weight of the fat pad. The white adipose tissue weight of the PG, VN, and combination group was significantly lower than that of the HF diet group (*P* < 0.05). In addition, the PG-VN combination group showed a significant difference compared with PG (*P* < 0.05).

### 3.2. Effects of PG, VN, and Their Combination on Lipid Level in HF Diet-Induced Obese C57BL/6J Mice

The blood plasma parameter changes are shown in Figures 1(d)–1(g). The level of total cholesterol in plasma is the major determinant of the risk of vascular disease, and lowering the LDL cholesterol level diminishes the risk of vascular diseases [32–34]. Furthermore, abdominal obesity is known to be associated with dyslipidemia, as characterized by increased triglyceride and decreased HDL cholesterol levels [35]. The total cholesterol level, triglyceride level, and LDL cholesterol level in the HF diet group were 2.8-fold, 2.5-fold, and 3-fold increased, respectively, compared with the standard chow diet group. The PG group, the VN group, and the PG-VN combination group all showed significant decreases in total cholesterol, triglyceride, and LDL cholesterol concentrations compared to the HF diet group (Figures 1(e)–1(g)). The HDL cholesterol level also showed an increase in the HF diet group compared with the standard chow diet group, but there were no significant differences in HDL cholesterol level between the HF diet group and the other four groups. In order to investigate any possible internal toxicity, the serum levels of alanine transaminase (ALT), aspartate transaminase (AST), blood urea nitrogen (BUN), and creatinine were evaluated and the results are shown in Supplementary Figures S1(a)–S1(d) (Supplementary Material available online at http://dx.doi.org/10.1155/2013/732126).

### 3.3. Effects of the Water Extract of PG on Adipogenesis and Transcription Factors in 3T3-L1 Cells

**Figure 2:**
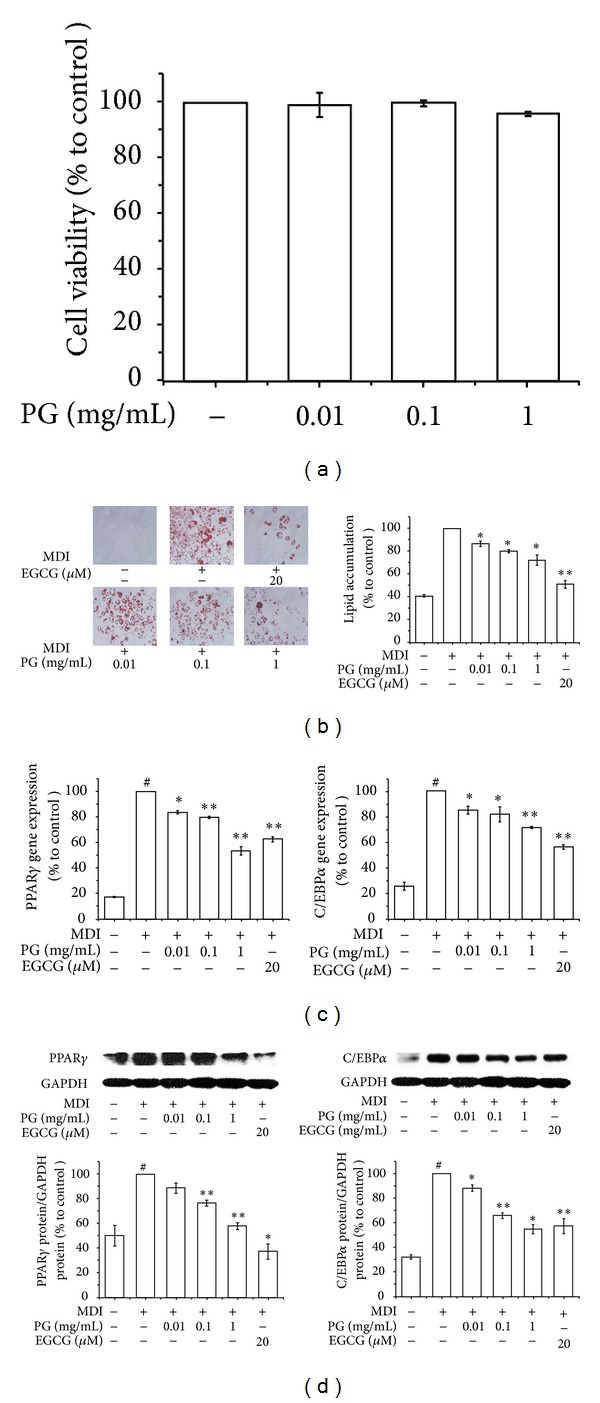
Effects of the water extract of PG on adipogenesis and transcription factors in 3T3-L1 cells. An MTS assay (a), Oil Red O staining assay (b), real-time RT-PCR assay (c), and Western blot assay (d) were performed to measure the effects of PG in 3T3-L1 cells. 3T3-L1 preadipocytes were induced to differentiate with 10% FBS/DMEM medium containing insulin, DEX, IBMX, and 0, 0.01, 0.1, or 1 mg/mL of PG extract for 2 days, and then the culture media were replaced with 10% FBS/DMEM medium containing insulin for the following 2 days. The control was treated with DW instead of extracts. EGCG was used as a positive control. Assays were performed in duplicates for each concentration, and experiments were repeated at least three times. Data represented are the relative expression. All values are mean ± SD. ^#^ *P* < 0.05, versus undifferentiated control cells; *P < 0.05 and **P < 0.005, versus differentiated control cells; DW, distilled water; PG, *Panax ginseng*; EGCG, epigallocatechin gallate.

Before investigating the antiobesity effects of PG *in vitro*, a cell viability test in 3T3-L1 preadipocytes was performed first. The MTS assay was performed to assess the effect of the water extract of PG on 3T3-L1 cell viability. As shown in Figure 2(a), the water extract of PG showed no significant effect on viability after 48 h treatment at concentrations of 0.01, 0.1, and 1 mg/mL. Further investigations were hence carried out at concentrations of 0.01, 0.1, and 1 mg/mL. Next, to investigate the effects of PG on preadipocytes differentiation, the lipid accumulation was measured by an Oil Red O staining assay. As shown in Figure 2(b), PG suppressed lipid accumulation in 3T3-L1 adipocytes in a dose-dependent manner with statistical significance (*P* < 0.05), suggesting that PG inhibits adipogenesis in 3T3-L1 cells. Epigallocatechin gallate (EGCG), a green tea compound that accounts for 54–59% of total tea catechins [36], was used as a positive control. Adipocyte differentiation accompanies the changes in expression of various adipogenic and lipogenic genes [37]. PPAR*γ* and C/EBP*α*, well-recognized adipogenic genes known to have roles in the early stage of adipogenesis [38], were examined. To investigate the inhibitory mechanism, the effects of PG on both mRNA and protein expression levels of PPAR*γ* and C/EBP*α* were examined. Fully differentiated 3T3-L1 cells were exposed for 48 h to concentrations of 0.01, 0.1, and 1 mg/mL of PG. Expression of both adipogenic genes PPAR*γ* and C/EBP*α* was significantly suppressed by PG in a dose-dependent manner. The results are shown in Figure 2(c). These results suggest that PG has inhibitory effects on PPAR*γ* and C/EBP*α*, which are found almost exclusively in adipose tissues and play crucial roles in the induction of adipose-specific genes and in the manifestation of the mature adipose phenotype [39]. In order to confirm the effects of PG on PPAR*γ* and C/EBP*α*, a Western blot analysis was carried out.  Figure 2(d) shows that the expressions of PPAR*γ* and C/EBP*α* significantly are suppressed by PG at concentrations of 0.1 and 1 mg/mL. Consistent with the results of the real-time RT-PCR assay shown above (Figure 2(c)), the Western blotting analyses confirm that PG has inhibitory effects on PPAR*γ* and C/EBP*α*. 

### 3.4. Effects of the Water Extract of VN on Adipogenesis and Transcription Factors in 3T3-L1 Cells

**Figure 3:**
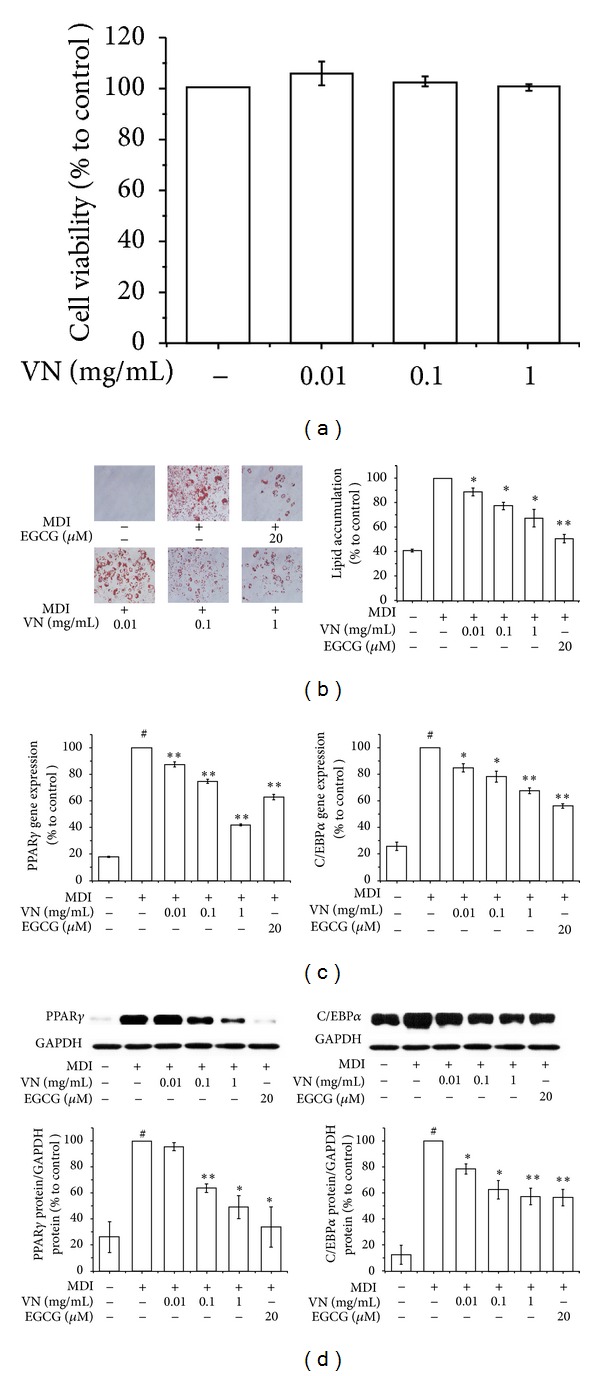
Effects of the water extract of VN on adipogenesis and transcription factors in 3T3-L1 cells. An MTS assay (a), Oil Red O staining assay (b), real-time RT-PCR assay (c), and Western blot assay (d) were performed to measure the effects of VN in 3T3-L1 cells. 3T3-L1 preadipocytes were induced to differentiate with 10% FBS/DMEM medium containing insulin, DEX, IBMX, and 0, 0.01, 0.1, or 1 mg/mL of VN extract for 2 days, and then the culture media were replaced with 10% FBS/DMEM medium containing insulin for the following 2 days. The control was treated with DW instead of extracts. EGCG was used as a positive control. Assays were performed in duplicates for each concentration, and experiments were repeated at least three times. Data represented are the relative expression. All values are mean ± SD. ^#^ *P* < 0.05, versus undifferentiated control cells; *P < 0.05 and **P < 0.005, versus differentiated control cells; DW, distilled water; VN, *Veratrum nigrum*; EGCG, epigallocatechin gallate.

Ahead of any other investigation, a cell viability assay of VN was performed as well as PG. As shown in Figure 3(a), an MTS assay verified that VN had no cytotoxic effects in 3T3-L1 cells at the concentrations of 0.01, 0.1, and 1 mg/mL. Further investigations were performed at the concentrations that did not show cytotoxicity, and EGCG was used as a positive control. The effect of VN on lipid accumulation in 3T3-L1 cells was measured via an Oil Red O staining assay. Similar to the results of PG, Figure 3(b) indicates that VN suppressed lipid accumulation in 3T3-L1 cells in a dose-dependent manner (*P* < 0.05). In a real-time RT-PCR assay, the results showed that VN also had significant inhibitory effects on the expression of PPAR*γ* and C/EBP*α* in a dose-dependent manner (Figure 3(c)). In addition, the results of the Western blot analysis implied that VN had inhibitory effects on expression of PPAR*γ* and C/EBP*α* in a dose-dependent manner as well as on the protein levels (Figure 3(d)).

### 3.5. Comparison of the Effects of the Mixture and the Combination Extract of PG and VN on Differentiation of 3T3-L1 Cells

**Figure 4:**
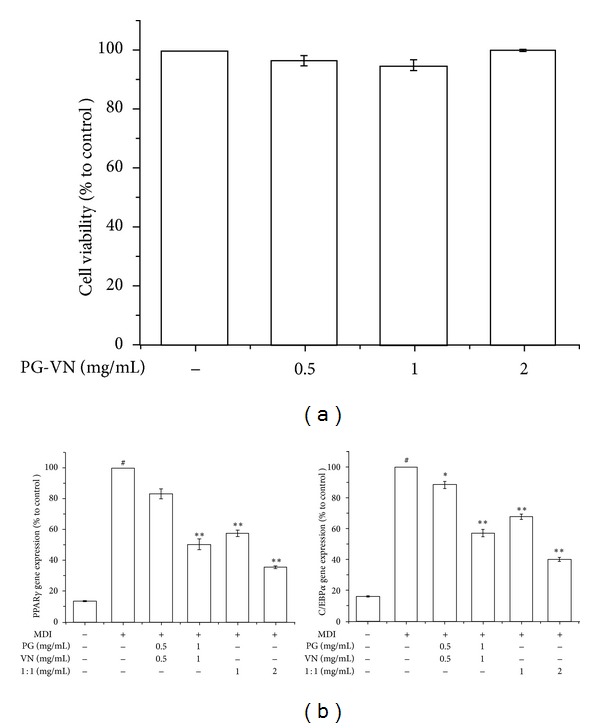
Comparison of the effects of the mixture and the combination extract of PG and VN on differentiation of 3T3-L1 cells. An MTS assay (a) was performed to measure the effects of PG-VN combination in 3T3-L1 cells. A real-time RT-PCR assay was performed to measure the difference between the effects of the combination and the mixture on PPAR*γ* and C/EBP*α* mRNA expressions in 3T3-L1 cells (b). 3T3-L1 preadipocytes were induced to differentiate with 10% FBS/DMEM medium containing insulin, DEX, IBMX, and 0, 0.01, 0.1, or 1 mg/mL of PG-VN mixture or PG-VN combination for 2 days, and then the culture media were replaced with 10% FBS/DMEM medium containing insulin for the following 2 days. The control was treated with DW instead of extracts. EGCG was used as a positive control. Assays were performed in duplicates for each concentration, and experiments were repeated at least three times. Data represented are the relative expression. All values are mean ± SD. ^#^ *P* < 0.05, versus undifferentiated control cells; *P < 0.05 and **P < 0.005, versus differentiated control cells; DW, distilled water; PG, *Panax ginseng*; VN, *Veratrum nigrum*; 1 : 1, PG-VN combination.

The cytotoxicity of the PG-VN combination extract was examined using an MTS assay. The MTS assay was performed to assess the effect of the water extract of the 1 : 1 PG-VN combination on 3T3-L1 cell viability. As seen in Figure 4(a), the water extract of the 1 : 1 PG-VN combination showed no significant effect on viability after 48 h treatment at concentrations of 0.5, 1, and 2 mg/mL. Because the highest concentration (2 mg/mL) of 1 : 1 combination extract did not have any toxic effects on 3T3-L1 adipocytes, further investigations were carried out at concentrations of 1 and 2 mg/mL, including PG and VN at a concentration of 1 mg/mL, respectively. Next, in order to identify the differences in adipogenic effects between the mixture (1 : 1 mixture of PG extract and VN extract) and the combination (1 : 1 PG and VN combination extract), a real-time RT-PCR analysis was performed to investigate whether the effects are clearly different. The mixture was prepared by mixing PG extract and VN extract at the same ratio, and the combination was prepared by water extraction of PG and VN as described above. The results of Figure 4(b) show that the combination formula of PG and VN had significantly higher effects on inhibition of PPAR*γ* and C/EBP*α* than the mixture of PG and VN, possibly providing scientific evidence supporting the prescription of a combination of herbs in KM. Due to this result, further investigations were performed using the PG-VN combination rather than the PG-VN mixture.

### 3.6. Synergistic Effects of PG-VN Combination Extract on Differentiation of 3T3-L1 Cells

**Figure 5:**
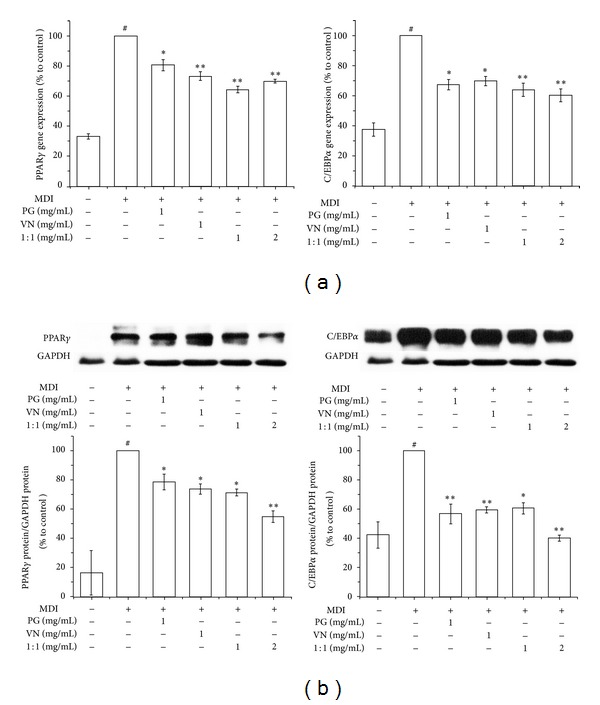
Synergistic effects of PG-VN combination extract on differentiation of 3T3-L1 cells. A real-time RT-PCR assay (a) and Western blot assay (b) were performed to measure the effects of PG-VN combination in 3T3-L1 cells. 3T3-L1 preadipocytes were induced to differentiate by 10% FBS/DMEM medium containing insulin, DEX, IBMX, and 0, 0.01, 0.1, or 1 mg/mL of PG-VN mixture or PG-VN combination for 2 days, and then the culture media were replaced with 10% FBS/DMEM medium containing insulin for the following 2 days. The control was treated with DW instead of extracts. EGCG was used as a positive control. Assays were performed in duplicates for each concentration, and experiments were repeated at least three times. Data represented are the relative expression. All values are mean ± SD. ^#^ *P* < 0.05, versus undifferentiated control cells; *P < 0.05 and **P < 0.005, versus differentiated control cells; DW, distilled water; PG, *Panax ginseng*; VN, *Veratrum nigrum*; 1 : 1, PG-VN combination.

Next, in order to compare the effects of PG and VN with their combination under the same conditions, a real-time RT-PCR assay was carried out. As seen in Figure 5(a), the expressions of PPAR*γ* and C/EBP*α* were significantly decreased by PG and VN. PG-VN combination at 1 and 2 mg/mL also showed a significant decrease compared with the control. PPAR*γ* expression of the PG-VN combination at 1 and 2 mg/mL was significantly decreased compared with PG.  Table 3 indicates that in both PPAR*γ* and C/EBP*α*, the PG-VN combination may have synergistic action and exhibits enhanced effects at a concentration of 1 mg/mL, since the inhibition rates of PG-VN combination (PPAR*γ*: 35.75 ± 2.07%, C/EBP*α*: 36.32 ± 4.34%) were higher than the sum of those of PG and VN at 1 mg/mL divided in half (PPAR*γ*: 23.195 ± 3.155%, C/EBP*α*: 31.92 ± 3.165%), but at higher dose, it did not show any synergism. Instead, the PG-VN combination inhibition rates at 2 mg/mL (PPAR*γ*: 30.19 ± 1.30%, C/EBP*α*: 39.88 ± 4.15%) were lower than the sum of the 1 mg/mL PG and VN inhibition rates (PPAR*γ*: 46.39 ± 6.31%, C/EBP*α*: 63.84 ± 6.33%). This suggests the possibility of antagonistic features at higher concentrations. In the case of C/EBP*α*, the difference in the inhibition rate (23.96 ± 10.48%) was larger than that in PPAR*γ* (16.20 ± 7.61%), and this indicates the antagonism at high concentration was expressed more strongly on C/EBP*α* than on PPAR*γ*.

To confirm the results shown above regarding protein levels, we compared the effects of PG and VN with their combination using a Western blotting assay. PG and VN at 1 mg/mL showed significant differences compared with the control in both PPAR*γ* and C/EBP*α* protein expressions (Figure 5(b)). In addition, the PG-VN combination also exerted a significant decrease on PPAR*γ* and C/EBP*α* expressions at two different concentrations (1 and 2 mg/mL). Similar to the mRNA expression results (Table 3), the results regarding protein level of both PPAR*γ* and C/EBP*α* (Table 4) also implied that the PG-VN combination may have synergistic interaction at the concentration of 1 mg/mL, since the inhibition rate of PG-VN combination on PPAR*γ* (28.87 ± 2.25%) was higher than the sum calculation of those on PPAR*γ* of PG and VN at 1 mg/mL divided in half (23.905 ± 4.375%), but the C/EBP*α* expression (39.61 ± 3.82%) showed antagonistic effects compared with the calculation (42.035 ± 4.44%). The PG-VN combination inhibition rates at 2 mg/mL (PPAR*γ*: 45.22 ± 3.88%, C/EBP*α*: 59.94 ± 1.94%) were lower than the sum of the 1 mg/mL PG and VN inhibition rates (PPAR*γ*: 47.81 ± 8.75%, C/EBP*α*: 84.07 ± 8.88%). The difference in the inhibition rate of C/EBP*α* (24.13 ± 10.82%) was larger than that of PPAR*γ* (2.59 ± 12.63%), suggesting that the antagonism at high concentration was expressed stronger on C/EBP*α* than on PPAR*γ*, supporting the former gene expression results.

## 4. Discussion

Many of the most effective phytomedicines are provided on the drug market as whole extracts of herbs, and practitioners have always believed that synergistic interactions between the components of individual or mixtures of herbs are a vital part of their therapeutic efficacy [40]. KM is characterized by the wide use of herbal formulas, which are capable of systematically treating disease determined by interactions among various herbs. However, the science underlying the combination rule of KM herbal formulas remains a mystery due to a lack of experimental evidence. Although some studies on the interaction between herbs have been performed, most focused on the synergistic interactions, that is, the “Sang-sa” or “Sang-o” relationship [40–46]; studies focusing on antagonism, that is, “Sang-ban” or ‘Sang-o,” have not yet been published. According to *Shennong's Classic of Materia Medica*, the classic of herbs written in 5 C, VN has a “Sang-ban” relationship with PG. This text does not specify the exact effects when used together, but every herb classic since *Shennong's Classic of Materia Medica* has forbidden usage of PG and VN together [12]. In this study, we investigated the antiobese effects of PG and VN combination, the representative example of “Sang-ban.” The effects of PG and VN extracts on obesity are investigated both *in vivo* and *in vitro*, and an investigation of the effects of their combination is followed.

Obesity is a chronic metabolic disorder caused by imbalance between energy intake and expenditure [47]. The prevalence of obesity is increasing in developed countries, and rates in the developing world are rapidly rising as well. The consequences of this are morbidity and mortality associated with other serious medical diseases, such as diabetes, hyperlipidemia, hypertension, cardiovascular diseases, osteoarthritis, and cancer [48]. Current medications for the treatment of obesity include mixed noradrenergic-serotonergic agents (sibutramine) [49] and absorption-reducing agents (orlistat) [50]. However, the usage is limited by their adverse effects. Sibutramine is known to increase blood pressure, which may cause cardiac arrhythmias, constipation, and headache with only minimum weight loss [49]. Orlistat, one of the most widely used obesity treatments, has also been reported to cause steatorrhea and deficiencies in lipid-soluble vitamins and essential fatty acids [50]. Furthermore, the first selective cannabinoid receptor CB1 blocker, rimonabant, was officially withdrawn from the market due to its serious side effects [51]. Interest in natural products for the treatment of obesity is thus rapidly growing. During the last decade, researchers have focused on the discovery of new drugs to reduce obesity, one of the most important health issues of modern society. 

In the present study, we have shown the effects of PG, VN, and their combination on obesity in HF diet-induced obese mice and in 3T3-L1 cells. PG, VN, and PG-VN combination significantly reduced weight gain and the fat pad weight in HF induced obese mice. They also significantly decreased lipid accumulation and the expressions of two major adipogenesis factors, PPAR*γ* and C/EBP*α*, in 3T3-L1 cells. Interestingly, the PG-VN combination had higher effects on PPAR*γ* and C/EBP*α* than the mixture of postextracted PG and VN. We found that the PG-VN combination had synergistic effects compared with a mixture of the extracts of PG and VN on inhibition of PPAR*γ* and C/EBP*α* expressions at lower doses. The PG-VN combination at 1 mg/mL showed stronger effects on gene and protein expressions of PPAR*γ* and C/EBP*α* than the calculated sum of separate PG and VN at 1 mg/mL divided into half. This result may constitute scientific evidence supporting the prescription of a combination of herbs instead of separate herb extraction mixtures in KM. However, because the exact mechanism was not fully revealed in this study, analytical assays such as HPLC should be carried out to investigate the componential differences between the combination and the mixture.

The study also suggests a potentially contentious issue that is unknown and at the same time could be a potential area of KM: the forbidden herb pairs, “Sang-ban,” known to have antagonistic effects when used together, introduced in *Shennong's Classic of Materia Medica*. The “Sang-ban” classification has been unquestioned until now, but the present study indicates the possibility of conflicting views. In *Shennong's Classic of Materia Medica*, the medical treatment of PG and VN pair is strictly forbidden [12]. But in this study on obesity, the PG and VN combination showed potential synergism. Obesity is a recently growing modern disease. In the past, weight loss may have been considered as an adverse effect, and this might partly explain the synergistic effects of PG-VN combination on obesity. However, further investigations must be followed. Although we have shown in this study that one of the representative “Sang-ban” pairs, PG and VN, shows synergism with regard to obesity, evidential verification of other pairs of the “Chil-jeong” theory is necessary.

## 5. Conclusion

In conclusion, the results of this study show the antiobese effects of PG and VN both *in vivo* and *in vitro*. PG, VN, and PG-VN combination significantly reduced weight gain and the fat pad weight in HF-induced obese mice. They also significantly decreased lipid accumulation and the expressions of two major adipogenesis factors, PPAR*γ* and C/EBP*α*, in 3T3-L1 cells. In addition, PG-VN combination had synergistic effects compared with the mixture of the extract of PG and VN on inhibition of PPAR*γ* and C/EBP*α* expressions at lower doses. These results show potential for a new pharmaceutical antiobese therapy and at the same time point toward a scientific basis for the usage of combinations in KM. Further investigations on combinations of various herbs should be carried out in future studies. 

## Supplementary Material

The serum levels of ALT, AST, BUN, and creatinine were detected to check any possible internal toxicity in HF diet-induced obese C57BL/6J mice. The PG-VN group did not show any particular toxicity to the liver or kidney compared to the PG group or VN group.

## Figures and Tables

**Table 1:** Composition of experimental diets (g/kg).

Compositions	Control	HFD*	PG*	VN*	1 : 1*	Slinti*
Casein	200.0	265.0	265.0	265.0	265.0	265.0
L-Cystine	3.0	4.0	4.0	4.0	4.0	4.0
Corn starch	397.486	—	—	—	—	—
Maltodextrin	132.0	160.0	160.0	160.0	160.0	160.0
Sucrose	100.0	90.0	90.0	90.0	90.0	90.0
Lard	—	310.0	310.0	310.0	310.0	310.0
Soybean oil	70.0	30.0	30.0	30.0	30.0	30.0
Cellulose	50.0	65.5	65.5	65.5	65.5	65.5
Mineral mix^a^	35.0	48.0	48.0	48.0	48.0	48.0
Calcium phasphate, dibasic	—	3.4	3.4	3.4	3.4	3.4
Vitamin mix^b^	10.0	21.0	21.0	21.0	21.0	21.0
Choline bitartrate	2.5	3.0	3.0	3.0	3.0	3.0
TBHQ, antioxidant^c^	0.014	—	—	—	—	—
Blue food color	—	0.1	0.1	0.1	0.1	0.1
PG	—	—	0.75	—	—	—
VN	—	—	—	0.75	—	—
PG-VN combination	—	—	—	—	0.75	—
Slinti	—	—	—	—	—	0.75

^a^Mineral mix, AIN-93G-MX (94046) containing (g/kg): calcium phosphate dibasic 500, sodium chloride 74, potassium citrate 220, potassium sulfate 52, magnesium oxide 24, manganous carbonate 3.5, ferric citrate 6, zinc carbonate 1.6, cupric carbonate 0.3, potassium iodate 0.0.1, sodium selenite 0.01, and chromium potassium sulfate 0.55. ^b^Vitamin mix, AIN-93-VX (94047), containing (g/kg): thiamin HCl 0.6, riboflavin 0.6, pyridoxine HCl 0.7, niacin 3, calcium pantothenate 1.6, folic acid 0.2, biotin 0.02, vitamin B12 (0.1 % trituration in mannitol) 1, dry vitamin A palmitate (500,00 U/g) 0.25, and menadione sodium bisulfite complex 0.15. ^c^TBHQ: tertiary butylhydroquinone. *60 % of total calories come from fat. HFD: high-fat diet; PG: *Panax ginseng*; VN: *Veratrum  nigrum; *1 : 1: PG-VN combination.

**Table 2:** Sequences of oligonucleotide primers (5′ to 3′) for real-time RT-PCR.

Genes	5′ to 3′ oligonucleotide sequences
Mouse PPAR*γ*	
Sense (forward)	TTT TCA AGG GTG CCA GTT TC
Antisense (reverse)	TTA TTC ATC AGG GAG GCC AG
Mouse C/EBP*α*	
Sense (forward)	GCC GAG ATA AAG CCA AAC AA
Antisense (reverse)	CCT TGA CCA AGG AGC TCT CA
Mouse GAPDH	
Sense (forward)	AAC TTT GGC ATT GTG GAA GG
Antisense (reverse)	GGA TGC AGG GAT GAT GTT CT

PPAR*γ*: peroxisome proliferator activated receptor *γ*; C/EBP*α*: CCAAT enhancer binding protein *α*; GAPDH: glyceraldehyde-3-phosphate dehydrogenase.

**Table 3:** Inhibition rate of PG, VN, and their combination on gene expression of PPAR*γ* and C/EBP*α*.

Treatment	PPAR*γ* (%)	C/EBP*α* (%)
PG 1 mg/mL	19.54 ± 3.62*	32.92 ± 3.34*
VN 1 mg/mL	26.85 ± 2.69**	30.92 ± 2.99*
PG-VN combination 1 mg/mL	35.75 ± 2.07^∗∗a^	36.32 ± 4.34**
PG-VN combination 2 mg/mL	30.19 ± 1.30^∗∗a^	39.88 ± 4.15**
^ x^PG + VN	46.39 ± 6.31	63.84 ± 6.33
^ x^(PG + VN)/2	23.195 ± 3.155	31.92 ± 3.165

Values were expressed as means ± SD. *P < 0.05, significantly different from the Control; **P < 0.005, significantly different from the control; ^a^ *P* < 0.05, significantly different from PG. ^x^Calculated additive response (the sum of the effects of the individual PG and VN treatments). Control, expression of the differentiated 3T3-L1 cells was considered as 0%; PG: *Panax ginseng*; VN: *Veratrum  nigrum.*

**Table 4:** Inhibition rate of PG, VN, and their combination on protein expression of PPAR*γ* and C/EBP*α*.

Treatment	PPAR*γ* (%)	C/EBP*α* (%)
PG 1 mg/mL	21.52 ± 5.28*	43.42 ± 6.80**
VN 1 mg/mL	26.29 ± 3.47*	40.65 ± 2.08**
PG-VN combination 1 mg/mL	28.87 ± 2.25*	39.61 ± 3.82*
PG-VN combination 2 mg/mL	45.22 ± 3.88^∗∗ab^	59.94 ± 1.94^∗∗b^
^ x^PG + VN	47.81 ± 8.75	84.07 ± 8.88
^ x^(PG + VN)/2	23.905 ± 4.375	42.035 ± 4.44

Values were expressed as means ± SD. *P < 0.05, significantly different from the Control; **P < 0.005, significantly different from the control; ^a^ *P* < 0.05, significantly different from PG; ^b^ *P* < 0.05, significantly different from VN. ^x^Calculated additive response (the sum of the effects of the individual PG and VN treatments). Control, expression of the differentiated 3T3-L1 cells was considered as 0%; PG: *Panax ginseng*; VN: *Veratrum  nigrum*.
