# Supplementary material for: Interaction of Veratrum nigrum with Panax ginseng against Obesity: A Sang-ban Relationship
Source: Evid Based Complement Alternat Med. 2013 Sep 2;2013:732126. doi: 10.1155/2013/732126 (PMC3773901; doi:10.1155/2013/732126)

Result Description

The serum levels of ALT, AST, BUN, and creatinine were detected to check any possible internal toxicity in HF diet-induced obese C57BL/6J mice. The PG-VN group did not show any particular toxicity to the liver or kidney compared to the PG group or VN group.

Figure Legend

SUPPLEMENTARY FIGURE 1: Effects of PG, VN, and their combinations on serum ALT, AST, BUN, and creatinine in HF diet-induced obese mice.

The serum levels of alanine transaminase (ALT) (a), aspartate transaminase (AST) (b), blood urea nitrogen (BUN) (c), and creatinine (d) were measured. All values are mean ± SD. $^{\#}p < 0.05$, significantly different from the Control; $^{*}p < 0.05$, significantly different from the HF diet group. Con, Standard laboratory diet group; HFD, high-fat diet group; PG, HF diet plus PG group; VN, HF diet plus VN group; 1:1, HF diet plus PG-VN combination group; Slinti, HF diet plus slinti group. PG, *Panax ginseng*; VN, *Veratrum nigrum.*

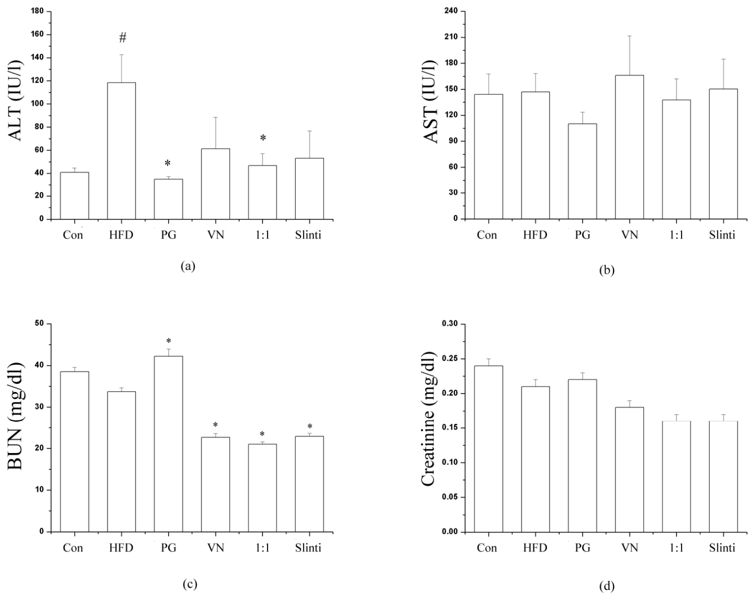

Supplement: Supplementary file 1 — The serum levels of ALT, AST, BUN, and creatinine were detected to check any possible internal toxicity in HF diet-induced obese C57BL/6J mice. The PG-VN group did not show any particular toxicity to the liver or kidney compared to the PG group or VN group. [file 732126.f1.pdf]
